# Histological Analysis of Oral Tissue Grafting: A Focus on Donor Site Selection

**DOI:** 10.3390/dj12090288

**Published:** 2024-09-10

**Authors:** Piero Antonio Zecca, Alice Ronchetti, Doris Cangelosi, Marcella Reguzzoni, Davide Farronato

**Affiliations:** 1 DIMIT, Department of Medicine and Technological Innovation, University of Insubria, 21100 Varese, Italy; 2 Private Practice, University of Insubria, 21100 Varese, Italy; 3 Private Practice, 20100 Milano, Italy

**Keywords:** histological analysis, oral tissue grafting, donor site selection, predictive modeling, gingival phenotypes

## Abstract

The science of dental tissue grafting is evolving, with an increased understanding of factors influencing graft behavior. Despite the widespread clinical use of soft tissue grafts, the histological characteristics of different gingival harvesting sites are still underexplored. This study aimed to fill this gap by analyzing 50 tissue samples harvested from 25 patients across three sites: the hard palate, maxillary tuberosity, and palatal rugae. Each sample underwent thorough histological and histomorphometric analysis. Conventional statistical analysis was performed using SPSS, while predictive modeling was conducted with RapidMiner Studio. The study identified significant histological differences among the graft sites, with notable variations in total graft height, epithelial height, and interdigitation perimeter. These findings underscore the importance of donor site selection in influencing graft success. Pair plots and principal component analysis (PCA) further highlighted the distinct histological features of each tissue type. The random forest classifier identified total graft height, epithelial height, and perimeter as the most influential factors in predicting graft site behavior. This study offers valuable insights into the histological characteristics of soft tissue grafts, potentially leading to more predictable clinical outcomes.

## 1. Introduction

Histological analysis plays a crucial role in understanding the behavior of dental tissue grafts. Connective tissue grafts are the gold standard for treating localized gingival recessions and other periodontal and peri-implant issues [1,2,3,4]. Despite the widespread use of these grafts, the qualitative analysis of grafts based on their harvesting sites remains underexplored, particularly their histological, morphometric, and cytological properties. Connective tissue grafts can be harvested from various intraoral sites, including the hard palate and maxillary tuberosity. Each site offers distinct advantages and poses unique challenges. The palate provides longer grafts, but with limited thickness due to the presence of vascular and neural structures [5,6,7].

In contrast, the maxillary tuberosity offers thicker grafts with minimal adipose and glandular tissue, which could enhance vascular perfusion and graft vitality [8,9]. Recent advancements in evidence-based medicine have shown that connective tissue from different oral mucosa areas exhibits distinct morphological characteristics. These findings underscore the importance of a detailed histological examination of grafts to predict surgical outcomes better.

The palatal rugae, anatomical folds on the anterior part of the hard palate, are another critical donor site for connective tissue grafts. These structures are known for their stable morphological characteristics. Clinically, palatal rugae grafts are favored for their relatively stable epithelial thickness and resilience to mechanical stress, which is essential for the longevity and functionality of the graft in dynamic oral environments [10]. Studies have shown that the tissue harvested from the palatal rugae exhibits unique histological features, such as a dense fibrous connective tissue matrix, which can contribute to the structural integrity and stability of the graft [11,12,13].

**Figure 1:**
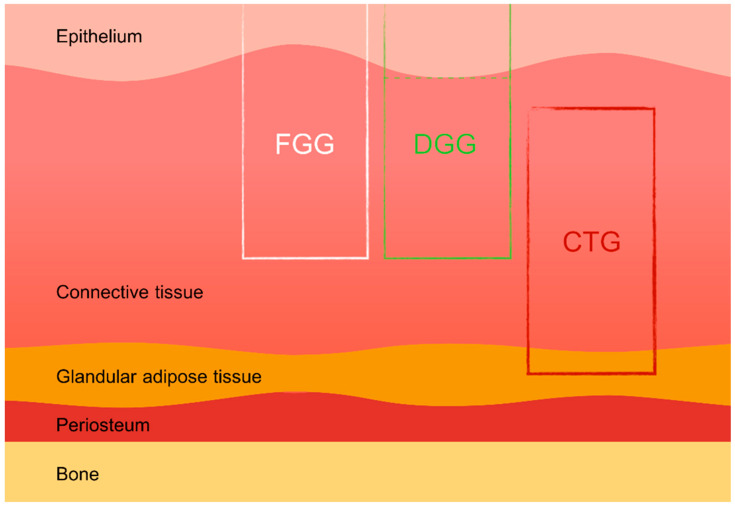
Types of palatal grafts. FGG—free gingival graft, DGG—de-epithelialized gingival graft, CTG—connective tissue graft.

Soft tissue grafts, including free gingival grafts (FGGs), subepithelial connective tissue grafts (SCTGs), and de-epithelialized gingival grafts (DGGs), are widely used in various dental procedures such as mucogingival surgery, root coverage techniques, peri-implant soft tissue augmentation, and socket preservation. These autologous grafts are essential for compensating soft and hard tissue deficiencies following tooth extraction, ultimately enhancing both the aesthetic and functional outcomes of implant-supported restorations [14,15]. Donor tissue for autografts can be harvested from several intraoral sites, most commonly the hard palate and the maxillary tuberosity. The palatal area allows more extended grafts to be harvested, while the maxillary tuberosity provides thicker grafts, which are particularly advantageous in cases requiring substantial tissue augmentation [16,17]. Studies have also indicated that tissues harvested from different areas of the palate (e.g., distal vs. mesial) exhibit varying histological characteristics, which can influence the success of the grafting procedure [18]. The scientific literature suggests that de-epithelialized gingival grafts (DGGs) offer superior long-term root coverage compared to subepithelial connective tissue grafts (SCTGs), with similar levels of donor site morbidity [2] (Figure 1).

Clinical evidence suggests that transplanted tissues tend to retain the morphological characteristics of their origin site, raising questions about the genetic determination of gingival biotypes versus their modulation through clinical intervention. “Phenotypic modulation” refers to the ability of donor tissues to influence and adapt to the characteristics of the recipient site, particularly in terms of tissue thickness and texture. For example, selecting donor sites with specific histological properties, such as thicker masticatory mucosa, can help achieve the desired outcome at the recipient site [19]. Short-term observations (3–6 months) of palatal grafts indicate stable initial volumes without discoloration [20]. However, grafts from the mesial area near the palatal rugae may develop an undulating morphology, reflecting the original tissue’s texture [21]. Long-term results demonstrate the tissue’s ability to adapt morphologically, guided by dental forms and prosthetic management. Grafts from the maxillary tuberosity show a propensity for hyperplastic growth, necessitating careful management of adjacent dental forms to limit excessive tissue growth. These grafts are particularly useful for compensating hard tissue deficiencies, but may present challenges due to variable outcomes influenced by surgical technique and genetic factors [22]. We aimed to fill this gap by conducting a monocentric prospective study to identify the histological characteristics of connective tissue grafts from different donor sites. By understanding the histological differences, we aim to improve the predictability and success of dental tissue grafting procedures.

## 2. Materials and Methods

This study was conducted at the Periodontal Department of the Dental Clinic, University of Insubria, enrolling 29 patients aged 18 and 45 years who required soft tissue grafts for various clinical indications, including gingival recession, soft tissue augmentation around dental implants, and socket preservation. The study protocol received approval from the Scientific Research Ethics Committee of the University of Insubria (approval 826), Varese, Italy, ensuring adherence to ethical standards in line with the Declaration of Helsinki. Written informed consent was obtained from all participants.

Eligibility criteria included patients over 18 with a healthy periodontium, a demonstrated need for soft tissue grafting, and good compliance, characterized by full mouth plaque and bleeding scores below 20%. Exclusion criteria encompassed individuals with a history of soft tissue augmentation in the target area, heavy smokers, and those with local or systemic conditions that could interfere with routine periodontal therapy.

**Figure 2:**
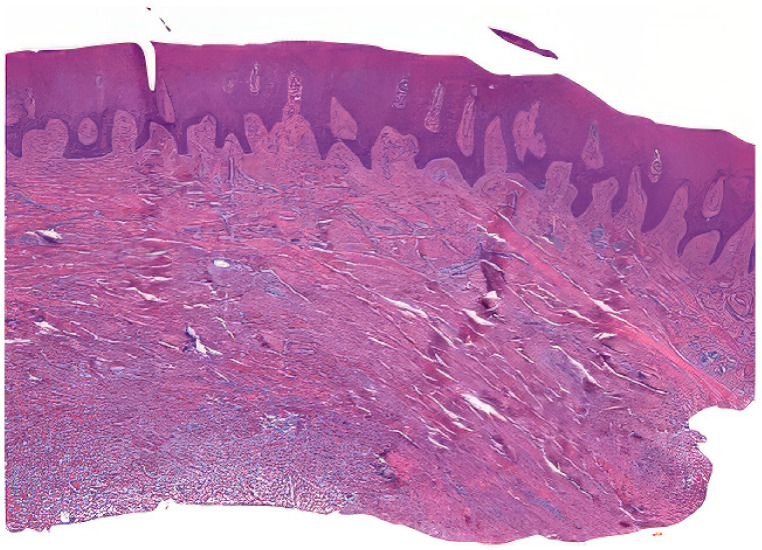
Example of a prepared sample.

Tissue samples were collected from excess epithelial–connective graft material, which underwent shaping and finishing procedures during surgery. A total of 50 samples from 25 patients were fixed in Karnovsky’s solution (2% paraformaldehyde, 2.5% glutaraldehyde in 0.1 M sodium cacodylate buffer) for 6 h at 4 °C, followed by washing in 0.1 M cacodylate buffer with added sucrose. After fixation in Karnovsky’s solution, the tissue samples were washed in 0.1 M cacodylate buffer with added sucrose to remove excess fixative. The dehydration process involved immersing the samples in increasing concentrations of ethanol (70%, 80%, 90%, 95%, and 100%), followed by clearing in xylene. The cleared samples were then infiltrated with paraffin at 60 °C under vacuum conditions to ensure thorough embedding. Serial sections of the embedded tissue blocks were cut at 4–5 μm thickness using a rotary microtome Leica SM 2400 microtome (Leica Biosystems, Nußloch, Germany). The sections were mounted on glass slides and dried at 37 °C overnight. Hematoxylin and eosin staining was performed (Figure 2) [23].

The stained sections were observed under a Nikon Eclipse 600 (Minato, Tokyo, Giappone) microscope with a Nikon DS-U1 digital sight camera. Two blinded expert operators (AZ and MR) performed the histological and histomorphometric analyses. NIS-Elements software 4.5 version facilitated measurements at low magnification (20×), focusing on parameters such as epithelial height, total graft height, and perimeter (Table 1).

Traditional statistical tests were used to analyze the data. The normality of the data distribution was assessed using the Shapiro–Wilk test, followed by one-way repeated-measure analysis of variance (ANOVA) for parametric data and the Kruskal–Wallis test for nonparametric data. Bonferroni correction was applied for multiple comparisons to ensure the reliability of the findings. The significance level was set at *p* < 0.05. Predictive analysis was conducted using RapidMiner Studio version 9.2 to identify patterns and correlations that might not be evident through conventional analysis alone. The predictive model used a random forest classifier to determine the most influential factors in predicting graft site behavior [24]. Principal component analysis (PCA) was also performed to visualize the separation between tissue types based on histological features.

## 3. Results

The analysis of the 50 tissue samples from 25 patients revealed significant variations in histological and histomorphometric characteristics across different graft sites. Notably, a significant difference in total graft height was observed between palatal rugae and tuber grafts (*p* < 0.001), with tuber grafts tending to hyperplastic growth. Similarly, the epithelial height differed significantly across the graft sites (*p* < 0.001), with palatal grafts maintaining a more stable epithelial thickness over time. The histological analysis revealed significant differences in the structural properties of the grafts from different donor sites. Palate samples exhibited higher epithelial–connective interdigitations and graft height values than palatal rugae and tuber. Specifically, the average total graft height was significantly greater in tuber grafts, indicating a propensity for hyperplastic growth (Table 2 and Table 3).

The results showed significant differences in tissue characteristics between graft sites by applying traditional statistical methods, including one-way repeated-measure analysis of variance and the Kruskal–Wallis test. Bonferroni correction was applied for multiple comparisons, ensuring the reliability of the findings.

These analyses underscored the significant differences in tissue characteristics between graft sites. The differences in epithelial height and total graft height suggest that the choice of donor site can impact the structural integrity and potential for successful graft integration.

The Kruskal–Wallis test results indicated that there were significant differences in the distributions of the measured parameters across the different harvesting sites (Table 4).

**Figure 3:**
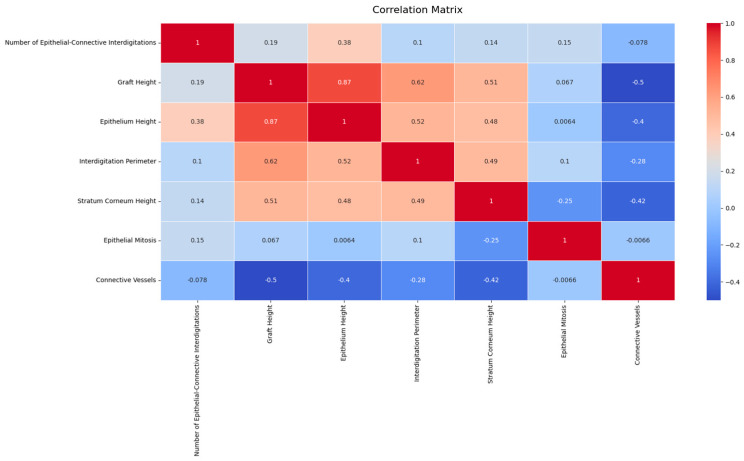
Histological patterns of palate, palatal rugae, and tuber tissues. The pair plot illustrates the distribution and relationships between key histological features across tissue types, showing distinct clustering.

The histological patterns of the different types (palate, palatal rugae, tuber) were analyzed using pair plots to visualize the distribution and relationships between variables. The pair plots revealed distinct clustering of the three types based on their histological features. For instance, palate samples exhibited higher epithelial–connective interdigitations and graft height values than palatal rugae and tuber. Similarly, the interdigitation perimeter and stratum corneum height significantly differed across the types (Figure 3).

**Figure 4:**
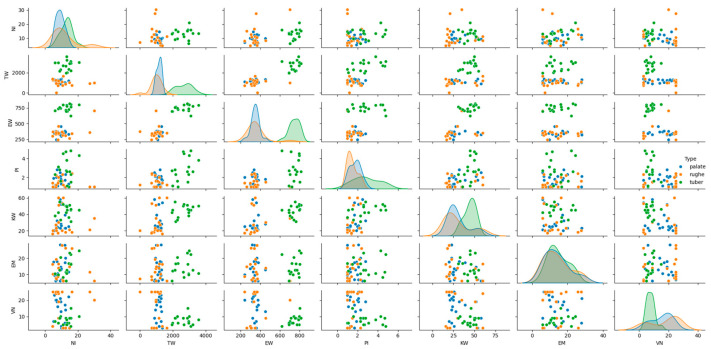
Confusion matrix and classification report for the random forest classifier, used to predict tissue types based on histological features.

The random forest classifier demonstrated good performance on the following metrics. The predictive accuracy of the model was high, with precision, recall, and F1- scores above 0.90 for all tissue types, indicating the robustness of the model in classifying the grafts accurately (Figure 4 and Table 5).

**Figure 5:**
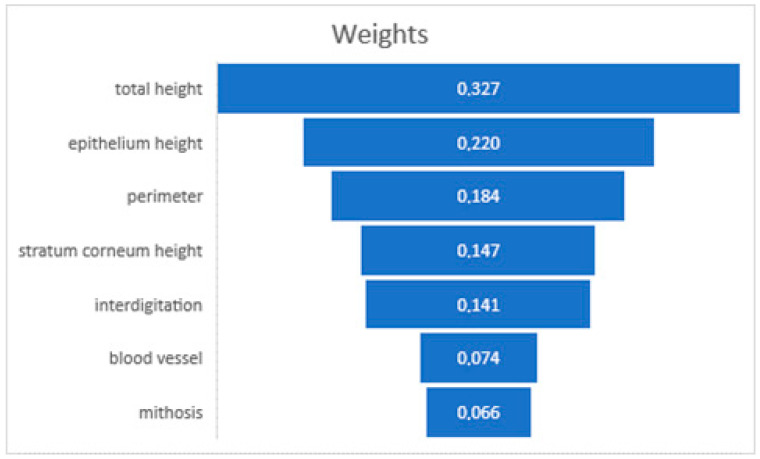
Decision-making weighting of AI software.

The analysis conducted using RapidMiner Studio showed that the weight factors of the prediction of the donor site for total graft height (32.7%), epithelial height (22%), and perimeter (18.4%) are the most influential factors in predicting graft site behavior [11] (Figure 5). This predictive model underscored the potential to enhance our understanding of tissue grafting, providing a data-driven basis for selecting optimal donor sites.

**Figure 6:**
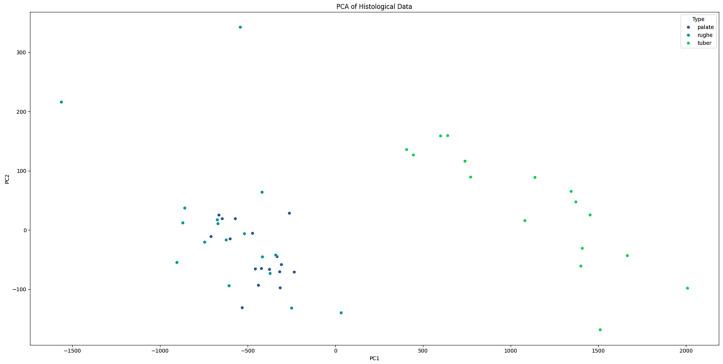
PCA plot showing the clear separation between “palate”, “palatal rugae”, and “tuber” tissue types based on histological features.

Additionally, principal component analysis (PCA) was performed to visualize the separation between tissue types based on their histological features. The PCA plot illustrated a clear separation between palate, palatal rugae, and tuber grafts, confirming the distinct histological characteristics of each type (Figure 6). The histological patterns of the different types (palate, palatal rugae, tuber) were analyzed using pair plots to visualize the distribution and relationships between variables. The pair plots revealed distinct clustering of the three types based on their histological features. For instance, palate samples exhibited higher epithelial–connective interdigitations and graft height values than palatal rugae and tuber. Similarly, the interdigitation perimeter and stratum corneum height significantly differed across the types.

## 4. Discussion

The present study aimed to provide a histological analysis of connective tissue grafts harvested from different donor sites, focusing on the hard palate, palatal rugae, and maxillary tuberosity. The findings reveal significant variations in these grafts’ histological and histomorphometric characteristics, which have implications for clinical practice in periodontal and peri-implant tissue augmentation.

The differences observed in the total graft height, epithelial height, and interdigitation perimeter among the donor sites underscore the importance of site selection in tissue grafting procedures. Tuber grafts exhibited the highest total graft height and epithelial height, suggesting greater potential for volumetric augmentation. This aligns with previous studies highlighting the hyperplastic growth tendency of tuber grafts, which can be advantageous for compensating hard tissue deficiencies but may require careful management to prevent excessive tissue growth [21,22].

Palatal grafts, particularly those harvested from the palatal rugae, demonstrated a more stable epithelial thickness. This stability is critical for ensuring the longevity and functionality of the graft, particularly in areas subjected to mechanical stress, such as during chewing. The interdigitation perimeter was also significantly greater in tuber grafts, indicating a more complex interface between the epithelium and connective tissue, which may enhance graft integration and stability.

The histological analysis revealed varying epithelial–connective interdigitations across the different graft sites. These interdigitations play a crucial role in the difficulty of obtaining a pure connective tissue graft. Specifically, more interdigitations make it challenging to follow the epithelial thickness and complete the graft’s de-epithelialization. In contrast, sites with fewer interdigitations allow for easier epithelium removal following its thickness, thereby facilitating the preparation of a pure connective tissue graft. In cases where interdigitations are numerous, a larger layer of tissue must be removed to ensure a safe margin and obtain a purely connective graft.

The predictive modeling using the random forest classifier identified total graft height, epithelial height, and interdigitation perimeter as the most influential factors in predicting graft site behavior. These findings suggest that these histological parameters can serve as reliable indicators for selecting the most appropriate donor site for specific clinical needs. The high accuracy of the model underscores its potential utility in clinical decision-making, providing a data-driven approach to optimize graft outcomes.

Principal component analysis (PCA) further validated the distinct histological characteristics of the different tissue types, with clear separation observed between palate, palatal rugae, and tuber grafts. This clear differentiation reinforces the concept that the choice of donor site can significantly influence the histological properties and consequently probably the clinical performance of the graft.

Our findings are consistent with those of Bertl et al., (2015) and Karring et al., (1975), who reported significant morphological variations in connective tissue grafts from different intraoral sites. The unique histological features of tuber grafts, such as increased epithelial height and complex interdigitations, have been previously noted for their potential to enhance graft integration [18,19].

However, our study provides a more comprehensive comparison, highlighting each donor site’s distinct advantages and challenges.

The stability of epithelial thickness observed in palatal rugae grafts aligns with the findings of Harris (2003), who emphasized the importance of maintaining epithelial integrity for successful graft outcomes. This stability is crucial for ensuring that the graft can withstand mechanical stresses and integrate seamlessly with the surrounding tissue.

Based on the histological and predictive modeling findings, several clinical recommendations can be made. For volumetric augmentation, especially in areas requiring substantial tissue thickness, tuber grafts are recommended due to their greater total graft height and complex interdigitations. However, clinicians should be vigilant about managing potential hyperplastic growth.

Palatal rugae grafts are preferable for applications requiring stable epithelial thickness and resistance to mechanical stress, such as root coverage procedures. These grafts offer a balance of adequate thickness and stability, making them suitable for areas subjected to functional loading.

This study has several limitations that should be addressed in future research. While the sample size was adequate for initial findings, it could be expanded to include a more diverse patient population to enhance the generalizability of the results. Additionally, while the predictive model demonstrated high accuracy, incorporating additional histological parameters and exploring other machine learning algorithms could further refine the model’s predictive capability.

Future research should also investigate the long-term clinical outcomes of grafts from different donor sites, correlating histological characteristics with functional and aesthetic results. This would provide a more comprehensive understanding of how histological properties influence clinical performance over time.

## 5. Conclusions

The most significant outcome of our study is the demonstration that the histological characteristics of palatal, rugae, and tuber tissues are distinct probably have crucial implications for the success of dental tissue grafting. The differentiation in total graft height, epithelial height, and interdigitation perimeter among these tissue types is not merely a statistical observation, but a fundamental insight into their biological behavior and integration potential.

While this study enhances our understanding of the inherent properties of each tissue type and highlights the potential for personalized approaches in tissue grafting, it also has certain limitations. Notably, the sample was relatively small, which may limit the generalizability of the findings. The study did not include stratification by patient characteristics such as sex, age, or other relevant factors like smoking status or systemic conditions, which could have influenced the results. The lack of stratification means that potential variations in tissue properties related to these factors were not fully explored, potentially overlooking important differences in graft behavior across patient demographics. Additionally, the study’s methodology primarily focused on histological and histomorphometric analysis without incorporating molecular techniques, such as immunohistochemistry or electron microscopy, which could provide a deeper understanding of the collagen types and other structural proteins in the grafts. This limitation could affect the precision with which graft characteristics are understood and their potential clinical outcomes.

Furthermore, though effective, the predictive model developed using machine learning was based on a limited set of histological parameters. The exclusion of other potential variables, such as clinical outcomes or long-term follow-up data, means that the model may not fully capture the complexity of factors influencing graft success. Expanding the model to include these additional variables could improve its predictive accuracy and clinical utility. Finally, the monocentric nature of the study, with all samples collected from a single geographical population, may restrict the results’ applicability to broader, more diverse populations. The absence of long-term follow-up also limits the ability to assess the durability and long-term success of the grafts, which are critical factors in determining the overall efficacy of tissue grafting procedures.

Our findings suggest that the choice of donor site can influence the outcome of the graft, thereby impacting the overall success of tissue grafting procedures.

This study not only helps us understand the inherent properties of each tissue type but also highlights the potential for personalized approaches in tissue grafting, where donor site selection is tailored to the specific needs of the recipient site.

One remaining question is how these histological insights can be integrated into routine clinical practice to improve graft success rates further.

Future research should aim to expand the sample size and include a more diverse patient population to improve the generalizability of the findings. Additionally, stratifying the analysis by factors such as sex, age, and other patient-specific characteristics could yield deeper insights into the variability in tissue properties. Furthermore, more detailed investigations into the collagen structure and type within the grafts, possibly through advanced techniques such as immunohistochemistry or electron microscopy, would provide a more comprehensive understanding of how these factors contribute to the success of tissue grafting.

## Figures and Tables

**Table 1:** Measured parameters.

Parameters	Abbreviations
Number of epithelial–connective interdigitations	NI
The perimeter of epithelial–connective interdigitations	PI
Maximum width of keratinized tissue	KW
Maximum width of the epithelium	EW
Total width of the sample;	TW
Epithelium mitosis	EM
Vessels number	VN

**Table 2:** Mean values and standard deviation.

Parameter	Palate	Palatal Rugae	Tuber
Total Graft Height (µm)	1200 ± 150	1000 ± 120	1500 ± 200
Epithelial Height (µm)	300 ± 50	250 ± 40	400 ± 60
Interdigitation Perimeter (µm)	500 ± 80	450 ± 70	600 ± 90

**Table 3:** One-way repeated measures.

Parameter	Test Statistic	*p*-Value	Adjusted *p*-Value
Total Graft Height	15.67	<0.001	<0.001
Epithelial Height	10.45	<0.001	<0.001
Interdigitation Perimeter	12.78	<0.001	<0.001

**Table 4:** Kruskal–Wallis test.

Epithelium Height vs. Site					
Harvesting Site	Test Statistics	Std. Error	Std. Test Statistics	Sig.	Adj. Sig.
palate vs. rugae	0.824	5000	0.165	0.869	1000
rugae vs. tuber	−25,136	5077	−4951	0.000	0.000
palate vs. tuber	−24,312	5077	−4788	0.000	0.000
**Interdigitations vs. Site**					
Harvesting site	Test statistics	Std. Error	Std. Test statistics	Sig.	Adj. Sig.
palate vs. rugae	−3824	5000	−0.765	0.444	1000
palate vs. tuber	−14,228	5077	−2802	0.005	0.030
rugae vs. tuber	−10,404	5077	−2049	0.040	0.243
**Total Height vs. Site**					
Harvesting site	Test statistics	Std. Error	Std. Test statistics	Sig.	Adj. Sig.
palate vs. rugae	6412	5000	1282	0.200	1000
rugae vs. tuber	−28,206	5078	−5555	0.000	0.000
palate vs. tuber	−21,794	5078	−4292	0.000	0.000
**Perimeter vs. Site**					
Harvesting site	Test statistics	Std. Error	Std. Test statistics	Sig.	Adj. Sig.
palate vs. rugae	6853	4982	1376	0.169	1000
rugae vs. tuber	−19,189	5059	−3793	0.000	0.001
palate vs. tuber	−12,336	5059	−2438	0.015	0.089
**Stratum Corneum Height vs. Site**					
Harvesting site	Test statistics	Std. Error	Std. Test statistics	Sig.	Adj. Sig.
palate vs. rugae	3176	4994	0.636	0.525	1000
rugae vs. tuber	−16,202	5072	−3194	0.001	0.008
palate vs. tuber	−13,026	5072	−2568	0.010	0.061

**Table 5:** Performance metrics for the random forest classifier in predicting tissue types based on histological features.

Type	Precision	Recall	F1 Score	Support
palate	1	0.83	0.91	6
palatal rugae	0.88	1	0.93	7
tuber	1	1	1	5
micro avg	0.95	0.95	0.95	18
macro avg	0.96	0.94	0.95	18
weighted avg	0.95	0.95	0.95	18

## Data Availability

Data are available on request from the authors.
